# Prevalence and determinants of academic bullying among medical and dental students in Jordan

**DOI:** 10.3389/frhs.2026.1932348

**Published:** 2026-08-25

**Authors:** Hana Taha, Omar Alshadaidah, Salwa Samhouri, Malek Awamleh, Hana Hadidi, Suhib Awamleh, Martin Söderberg, Vanja Berggren, Linus Jönsson

**Affiliations:** 1 Department of Family and Community Medicine, School of Medicine, The University of Jordan, Amman, Jordan; 2 Department of Neurobiology, Care Sciences and Society, Karolinska Institutet, Stockholm, Sweden; 3 Department of Internal Medicine, King Hussein Cancer Center, Amman, Jordan; 4 School of Dentistry, University of Jordan, Amman, Jordan

**Keywords:** academic bullying, Jordan, medical education, mental health, students' wellbeing

## Abstract

**Background:**

Academic bullying is a well-recognized issue in medical education, carrying considerable consequences for mental health, academic performance, and even patient care. The issue remains understated, as most victims refrain from reporting due to fear of repercussions. This is especially relevant in the MENA region, where data on the topic is already sparse. This study aims to report the prevalence, types, associated factors, and perceived effects of academic bullying among medical and dental students at a Jordanian university.

**Methods:**

A cross-sectional study collected the data from 383 medical and dental students at a public Jordanian university, using a self-administered questionnaire assessing sociodemographic, bullying involvement, types, determinants and responses. Depressive and anxiety symptoms were measured using the PHQ-9 and GAD-7. Victims and non-victims were compared using the Mann–Whitney U and chi-square tests; binary logistic regression identified independent predictors of victimization.

**Results:**

Of the 383 participants, 38.6% reported some involvement in bullying: 27.7% as witnesses only, 4.2% as victims only, and 6.8% as both victims and witnesses. Verbal bullying was the most prevalent form in both the witnessed and victimized groups (69.7% and 73.8%, respectively), with fellow students being the most commonly reported perpetrators. Independent predictors of victimization included a history of childhood bullying (OR = 12.05, 95% CI: 4.88–37.0) and lower GPA (OR = 0.38, 95% CI: 0.18–0.79)

**Conclusion:**

Academic bullying remains prevalent yet underreported among Jordanian medical and dental students, with measurable consequences for mental health and academic life. Certain groups — particularly those with a childhood bullying history —appear more vulnerable to victimization. These findings support the need for accessible, confidential reporting systems, routine mental health screening, and peer-support programs in medical education.

## Introduction

Violence, as defined by the World Health Organization, is the intentional use of physical force or power, whether threatened or actual, against oneself, another person, or a group or community, that results in or has a high likelihood of resulting in injury, death, psychological harm, maldevelopment, or deprivation (1). Within this framework, violence is commonly classified into three categories: self-directed, interpersonal, and collective violence, with interpersonal violence referring to harmful acts inflicted by an individual or a small group upon another person (1). Bullying is increasingly recognized as one manifestation of interpersonal violence, occurring most commonly among children, adolescents, and young adults within peer and educational settings (2).

Academic bullying is a specific manifestation of interpersonal violence within educational and training settings, defined by three core features that distinguish it from isolated mistreatment or interpersonal conflict: repeated negative actions, an intention to harm, and an imbalance of power between perpetrator and victim that is often reinforced by academic hierarchies (3). In medical education, bullying and mistreatment span a broad spectrum, including verbal abuse, discrimination, sexual advances, biased evaluations, and microaggressions (4, 5). Such mistreatment is frequently normalized within medical training as an expected part of the educational hierarchy, a perception that contributes to both its persistence and its underreporting.

Bullying has many negative consequences for affected students, particularly for mental health; reported rates include anxiety (29.5%) and depression (8.3%), and microaggressions correlate positively with depression (5, 6). More severe outcomes have also been reported, including suicidal ideation and attempts among mistreated students, alongside an increased prevalence of substance abuse (7–9). Mistreatment has further been identified as the strongest predictor of burnout, with bullied students showing higher exhaustion and disengagement scores and more career regret than their peers (10).

The impact of mistreatment and bullying extends beyond the mental health aspects and affects even the academic and educational experience for mistreated students, as notably 42% of students report disengagement from learning activities due to mistreatment (5). Additionally, students who experienced microaggressions at least once weekly were more likely to transfer schools, which points to a broader academic toll of such experiences (14.5% vs. 4.7%, *p* < 0.001) (6).

The consequences of mistreatment and bullying are not limited only to the individual level but extend further to professional relationships and patient care, as studies showed that 26% of providers reported that mistreatment harmed their communication with other team members, while 30% expressed fears and concerns that their past experiences of mistreatment would affect their communication in the future with other team members (11). Such disruptions to team dynamics can compromise patient care and safety.

In the United States, the prevalence of bullying and mistreatment among medical students is reported at 35.4%, based on AAMC Graduation Questionnaire data for the 2016–2017 graduating cohort, with verbal abuse and public humiliation the most commonly reported forms (12). These figures likely underestimate the true burden, as students often refrain from reporting due to fear of academic repercussions, uncertainty about reporting channels, or the belief that no action will follow. Sources of bullying and mistreatment vary, but in the clinical setting, residents, followed by faculty, are the most common sources (8) whereas in the preclinical setting, classmates account for the majority (about 87%), with the classroom the most common site (5).

Data from the wider Middle East and North Africa region are similarly limited, leaving the scope of the problem among Arab medical students largely uncharacterized. Recent studies from neighboring countries illustrate the scale of the problem, with reported bullying prevalence among medical students ranging from 11.5% at a Middle Eastern university (5) to 71.1% in Egypt (13). Data on bullying and harassment among medical students in Jordan, however, remain scarce, with most local studies focusing on nursing students. One study found that 20.4% of nursing students were victims of cyberbullying (14). Another reported that the most harassed nursing students were females, with verbal or psychological harassment the most common type (15). However, these studies have not discussed the negative outcomes nor the prevalence of bullying or harassment for medical students in Jordan. Medical and dental training in Jordan is shaped by a strongly hierarchical, seniority-oriented culture in which deference to instructors and senior clinicians is expected, and demanding or critical behavior toward juniors may be normalized as part of the educational process (16). Prevailing social norms that discourage the questioning of authority, together with the stigma attached to being seen as a complainer, may influence both how bullying occurs and whether students recognize and report it (17).

## Methods

### Study design

This was a cross-sectional descriptive study carried out between January and February 2026. The design was chosen to describe the prevalence, types, and perceived effects of academic bullying among medical and dental students at a single point in time, and because it is a practical approach for gathering baseline data on students' awareness of and attitudes toward bullying.

### Study setting

Medical education in Jordan has expanded over the past few decades, and the country now has six public and two private medical schools, with dental programs running alongside them. There are approximately 10,000 medical students nationally, with around 1,500 graduating each year (17). This study was conducted at the faculties of medicine and dentistry of a public university in Jordan. Students from all academic years, across both the basic science and clinical phases of training, were eligible to participate.

### Measurement tool

Data were collected using a structured, self-administered questionnaire developed by the research team through a review of the existing literature on academic bullying and iterative discussion among the investigators. Several items were adapted from instruments used in earlier bullying studies (5, 13) and were edited and supplemented with questions written specifically for this study. The questionnaire opened with a short introduction that defined academic bullying as repeated, unwanted, and aggressive behavior involving a real or perceived power imbalance within the educational setting, and that described its main forms (verbal, physical, social or relational, cyber, academic, and discriminatory) (18). This introduction was presented in both Arabic and English, so the concept was clear to every participant, while the questionnaire items themselves were administered in English.

The questionnaire was made up of five sections. The first collected sociodemographic and background information through 14 items, including age, gender, nationality, academic major, year of study, grade point average, socioeconomic status, birth order, family structure, and lifestyle factors such as smoking and caffeine consumption. The second and third sections used two validated screening tools: the Patient Health Questionnaire-9 (PHQ-9) (19) for depressive symptoms and the Generalized Anxiety Disorder-7 scale (GAD-7) (20) for anxiety symptoms.

The fourth section contained three questions on the participant's current use of antidepressants, anxiolytics, or psychotherapy. The fifth section assessed bullying directly: whether the student had been a victim, a witness, or a perpetrator, along with the type and frequency of bullying, the identity of the perpetrators, its academic and personal impact, how the participant responded, and their attitudes toward bullying.

Content and face validity were assessed by a panel of experts in the field, whose feedback was used to refine the wording, relevance, and comprehensiveness of the items and to reword or remove ambiguous items. The questionnaire was then pilot tested on a sample of 10 students from the target population to confirm its clarity, comprehensibility, and relevance, and minor revisions were made based on their feedback before data collection began. Responses from the pilot participants were excluded from the final analysis. Internal consistency was acceptable across the instrument, with Cronbach's alpha values of 0.86 for the PHQ-9 items, 0.89 for the GAD-7 items, and 0.78 for the bullying and attitudes items, all above the conventional threshold of 0.70.

### Sample size and data collection

The minimum required sample size was calculated using the Raosoft sample size calculator (21), assuming a 50% expected response distribution and an estimated source population of 6,500 undergraduate medical and dental students, with a 5% margin of error and a 95% confidence level, which gave a minimum of 363 participants. A total of 383 students were included in the final analysis, exceeding the required minimum.

Participants were recruited through convenience sampling, which was a feasible and cost-effective way to reach students at the university. The questionnaire was created on Google Forms and distributed online through official student WhatsApp and Facebook groups. The inclusion criteria were full-time undergraduate medical and dental students enrolled at the university across all academic years. Students who did not provide consent were excluded.

### Ethical considerations

The study was approved by the Institutional Review Board of the participating public university (approval no. 606/2026). All participants provided informed consent before taking part, and participation was voluntary and anonymous. No identifying information was collected, and the data were handled confidentially and used only for research purposes.

### Statistical analysis

Data were analyzed using R (version 4.5). Categorical variables are described using frequencies and percentages, while continuous variables are expressed as mean (standard deviation) or median (interquartile range) depending on the distribution. The Shapiro–Wilk test was used to assess normality of PHQ-9 and GAD-7 scores, both of which were found to be non-normally distributed (*p* < 0.001), therefore non-parametric tests were applied throughout.

Participants who did not consent (*n* = 10) were excluded from all analyses. Those who reported engaging in bullying as a perpetrator were also excluded (*n* = 9) due to small sample size and conceptual differences from the other groups, except when reporting their characteristics. The final analytical sample consisted of 383 participants. For the purpose of comparisons, participants were categorized as victims (those who reported being victimized, either alone or alongside witnessing, *n* = 42) and non-victims (*n* = 341).

Group comparisons between victims and non-victims were conducted using the Mann–Whitney U test for continuous variables and chi-square test with simulated *p*-values for categorical variables. Fisher's exact test was used when expected cell counts were small, particularly for treatment use variables. Multi-select survey questions (type of bullying, perpetrator identity) were split into binary dummy variables, one per response option, and frequencies were calculated independently.

Depression and anxiety were assessed using the Patient Health Questionnaire-9 (PHQ-9) and the Generalized Anxiety Disorder-7 scale (GAD-7). Total scores were computed by summing item responses coded 0–3. PHQ-9 severity was categorized as: a score of 10 or above was used as the threshold for probable depression and a GAD-7 score of 10 or above for probable anxiety.

Binary logistic regression was performed to identify factors associated with victimization. Predictors included gender, major, year of study, socioeconomic status, GPA, childhood bullying history and single parent family status. Due to missing data on predictor variables, the logistic regression was conducted on 352 of the 383 participants (complete case analysis). Multicollinearity was assessed using the variance inflation factor (VIF), all values were below 2 indicating no significant multicollinearity. Statistical significance was set at *p* < 0.05.

## Results

### Sample characteristics

A total of 383 students were included in the final analysis, after excluding participants who did not consent and perpetrators. The mean age was 20.8 years (SD = 1.7) and the majority were female (71.8%). Students were almost equally distributed between Medicine (52.2%) and Dentistry (47.8%). Most participants were in their second or third year of study (23.0% and 32.6% respectively). The mean GPA was 3.4 out of 4 (SD = 0.5) and mean BMI was 23.2 (SD = 4.3). A large proportion of students, 80.9%, held Jordanian nationality. Nearly half (42.0%) reported a history of bullying during childhood or school years, and 10.7% came from single parent families. Full sample characteristics are presented in Table 1.

**Table 1:** Sample characteristics (*N* = 383).

Characteristic	*N* = 383
Age, mean (SD)	20.8 (1.7)
Gender	
Female	275 (71.8%)
Male	108 (28.2%)
Major	
Medicine	200 (52.2%)
Dentistry	183 (47.8%)
Year of study	
1st	41 (10.7%)
2nd	88 (23.0%)
3rd	125 (32.6%)
4th	87 (22.7%)
5th	25 (6.5%)
6th	17 (4.4%)
Socioeconomic status	
Lower/lower-middle class	20 (5.2%)
Middle class	160 (41.8%)
Upper-middle class	175 (45.7%)
Upper class	28 (7.3%)
GPA (out of 4), mean (SD)	3.4 (0.5)
BMI, mean (SD)	23.2 (4.3)
Nationality	
Jordanian	310 (80.9%)
Non-Jordanian	73 (19.1%)
Birth order	
First child	122 (31.8%)
Middle	184 (48.0%)
Youngest	72 (18.8%)
Only child	5 (1.3%)
Single parent family	41 (10.7%)
Childhood bullying history	161 (42.0%)
Smoker (cigarettes or hookah)	58 (15.1%)
Drinks coffee	281 (73.4%)

Mean (SD) for continuous variables; *n* (%) for categorical variables.

### Prevalence and nature of bullying

Of the 383 students, 235 (61.4%) reported no involvement in bullying whatsoever. A total of 106 students (27.7%) witnessed bullying without being victimized themselves, while 16 (4.2%) reported being victims only and 26 (6.8%) were both victims and witnesses. For the purpose of further analysis, victims (*n* = 42) and witnesses (*n* = 132) were defined to include those in the overlap category.

Verbal bullying was the most common type reported, by 73.8% of victims and 69.7% of witnesses. Academic bullying was the second most frequent (42.9% and 48.5% respectively), followed by social/relational bullying (35.7% victims, 32.6% witnesses). Physical bullying was the least common, reported by 9.5% of victims and 17.4% of witnesses. Fellow students were the most frequently identified perpetrators in both groups: 61.9% among victims and 75.8% among witnesses. Lecturers or supervisors were implicated by 45.2% of victims and 39.4% of witnesses. Full breakdown of bullying type and perpetrators is presented in Table 2.

**Table 2:** Bullying characteristics.

Bullying role — full sample (*N* = 383)	n (%)	n (%)
No involvement	—	235 (61.4%)
Witness only	—	106 (27.7%)
Victim only	16 (4.2%)	—
Victim & witness	26 (6.8%)	—

**Type of bullying** ^a^	**Victim (*n*** **=** **42)**	**Witness (*n*** **=** **132)**
	***n* (%)**	***n* (%)**
Verbal	31 (73.8%)	92 (69.7%)
Academic	18 (42.9%)	64 (48.5%)
Social/relational	15 (35.7%)	43 (32.6%)
Cyber	8 (19.0%)	32 (24.2%)
Discriminatory	5 (11.9%)	23 (17.4%)
Physical	4 (9.5%)	23 (17.4%)
Perpetrator^a^	*n* (%)	*n* (%)
Fellow students	26 (61.9%)	100 (75.8%)
Lecturers/supervisors	19 (45.2%)	52 (39.4%)
Faculty members	8 (19.0%)	34 (25.8%)
Patients/family members	2 (4.8%)	8 (6.1%)

a Multiple selections allowed; percentages exceed 100%.

Witness group includes witness only (*n* = 106) and victim & witness (*n* = 26).

Victim group includes victim only (*n* = 16) and victim & witness (*n* = 26).

### Mental health outcomes

Among the full sample, the mean PHQ-9 score was 9.6 (SD = 6.2) with a median of 9. Probable depression was found in 174 students (45.4%). For anxiety, the mean GAD-7 score was 8.1 (SD = 5.7), and 136 participants (35.5%) met the threshold for probable anxiety. Regarding treatment, 18 students (4.7%) were on antidepressants, 27 (7.0%) were taking anxiolytics, and 23 (6.0%) were receiving psychotherapy at the time of the survey.

Victims had significantly higher GAD-7 scores compared to non-victims (median 10 vs. 7, *p* = 0.027). Although PHQ-9 scores were also higher in the victim group (median 10 vs. 9), this difference did not reach statistical significance (*p* = 0.140). Similarly, probable depression rates were higher among victims (52.4% vs. 44.6%) but the difference was not significant (*p* = 0.433). Probable anxiety however, was significantly more common in victims: 52.4% versus 33.4% in non-victims (*p* = 0.017).

**Figure 1:**
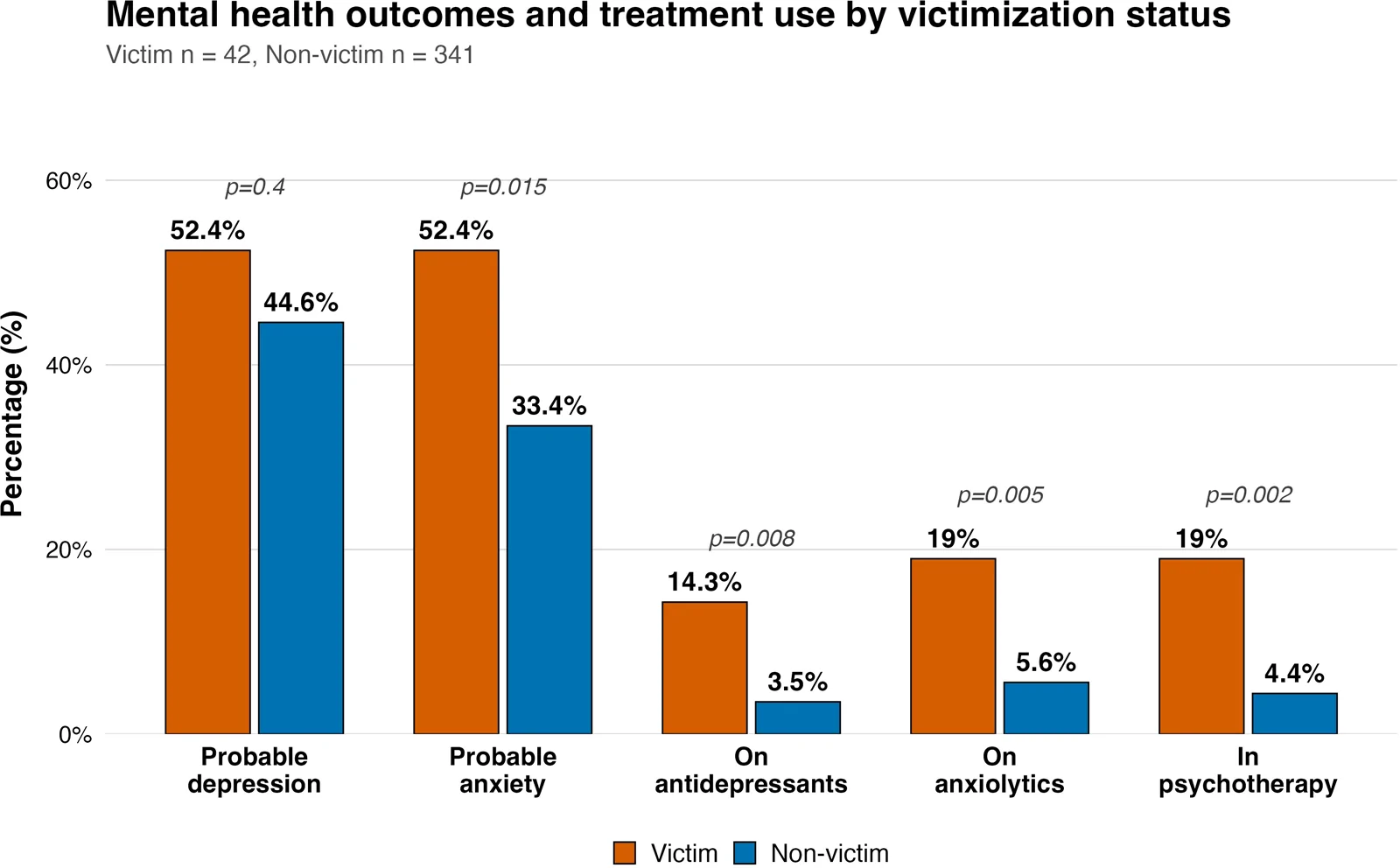
Mental health outcomes and treatment use by victimization status.

Victims were significantly more likely to be using mental health treatment. Antidepressant use was reported by 14.3% of victims versus only 3.5% of non-victims (*p* = 0.008). Anxiolytic use was also higher in victims (19.0% vs. 5.6%, *p* = 0.005), as was psychotherapy (19.0% vs. 4.4%, *p* = 0.002). These findings are illustrated in Figure 1 and detailed in Table 3.

**Table 3:** Comparison of victim and non-victim groups.

Variable	Overall *N* = 383	Victim *n* = 42	Non-victim *n* = 341	*p*-value
Demographics				
Age, median (IQR)	20 (20,22)	21 (20,22)	20 (20,21)	0.069
GPA, median (IQR)	3.5 (3,3.8)	3.2 (3,3.5)	3.5 (3,3.8)	0.001
BMI, median (IQR)	22.4 (20,25)	23.4 (22,26)	22.3 (20,25)	0.070
Female	275 (71.8%)	29 (69.0%)	246 (72.1%)	0.727
Medicine	200 (52.2%)	21 (50.0%)	179 (52.5%)	0.867
Childhood bullying history	161 (42.0%)	37 (88.1%)	124 (36.4%)	<0.001
Single parent family	41 (10.7%)	8 (19.0%)	33 (9.7%)	0.074
Smoker	58 (15.1%)	5 (11.9%)	53 (15.5%)	0.642
Mental health outcomes				
PHQ-9 score, median (IQR)	9 (5,13)	10 (7,16)	9 (4,13)	0.140
Probable depression	174 (45.4%)	22 (52.4%)	152 (44.6%)	0.4
GAD-7 score, median (IQR)	7 (4,12)	10 (7,16)	7 (4,11)	0.027
Probable anxiety	136 (35.5%)	22 (52.4%)	114 (33.4%)	0.015
Treatment use				
On antidepressants	18 (4.7%)	6 (14.3%)	12 (3.5%)	0.008
On anxiolytics	27 (7.0%)	8 (19.0%)	19 (5.6%)	0.005
In psychotherapy	23 (6.0%)	8 (19.0%)	15 (4.4%)	0.002

Median (IQR) for continuous variables; *n* (%) for categorical.

Mann-Whitney U test for continuous; chi-square with simulated *p*-value for categorical.

Probable depression = PHQ-9 ≥ 10. Probable anxiety = GAD-7 ≥ 10.

### Victim and witness behaviors

Among the 42 victims, verbal bullying was the most common type (73.8%), followed by academic (42.9%) and social/relational (35.7%). Fellow students were the most frequent perpetrators (61.9%), with lecturers or supervisors implicated by 45.2%. Bullying was most often rare or occasional in pattern (33.3% each) and exactly half reported an academic impact. The majority tried to intervene (61.9%), mainly by verbally asking the bully to stop (57.7%). Among those who did not (*n* = 16), the main reasons were avoiding trouble or walking away (56.2%) and fear of the bully's strength or backing (31.2%). Only 23.8% formally reported to a faculty member, though 66.7% told a parent or friend. Social support was rated a median of 3 out of 5, and 31.0% said the bullying was still ongoing. Bullying most frequently started in the first year of study (47.6%).

In the witness group (*n* = 132), the profile of bullying was largely comparable. Verbal bullying was again the most witnessed type (69.7%), and no significant difference was found in overall bullying type distribution (*p* = 0.800) or perpetrator profile (*p* = 0.715) between groups. Fellow students were the most frequently witnessed targets (76.5%), followed by friends (41.7%). The pattern of bullying did not differ significantly (*p* = 0.813). Witnesses were somewhat less likely to try and stop the bullying compared to victims (43.2% vs. 61.9%, *p* = 0.051). Reasons for not intervening (*p* = 0.078) and method of intervention (*p* = 0.076) also did not differ significantly, though “none of my business” was cited exclusively by witnesses (26.7% vs. 0%). Reporting rates (*p* = 1.000), telling parents or friends (*p* = 0.461), and ongoing bullying (*p* = 0.838) were all comparable between groups. Full data are in Table 4.

**Table 4:** Bullying characteristics and behavioral responses among victims and witnesses.

Variable	Victim *n* = 42	Witness *n* = 132	*p*-value
Type of bullying^a^	*n* (%)	*n* (%)	0.800
Verbal	31 (73.8%)	92 (69.7%)	
Academic	18 (42.9%)	64 (48.5%)	
Social/relational	15 (35.7%)	43 (32.6%)	
Cyber	8 (19.0%)	32 (24.2%)	
Discriminatory	5 (11.9%)	23 (17.4%)	
Physical	4 (9.5%)	23 (17.4%)	
Perpetrator^a^	*n* (%)	*n* (%)	0.715
Fellow students	26 (61.9%)	100 (75.8%)	
Lecturers/supervisors	19 (45.2%)	52 (39.4%)	
Faculty members	8 (19.0%)	34 (25.8%)	
Patients/family members	2 (4.8%)	8 (6.1%)	
Target of bullying^a^ (witnessed)	—	*n* (%)	
Fellow students	—	101 (76.5%)	
Friends	—	55 (41.7%)	
Faculty members	—	10 (7.6%)	
Lecturers	—	9 (6.8%)	
Patients/family members	—	4 (3.0%)	
Pattern of bullying	*n* (%)	*n* (%)	0.813
One-time incident	10 (23.8%)	31 (23.5%)	
Rare occurrence	14 (33.3%)	40 (30.3%)	
Occasional occurrence	14 (33.3%)	41 (31.1%)	
Frequent occurrence	2 (4.8%)	15 (11.4%)	
Recurring pattern	2 (4.8%)	5 (3.8%)	
Academic impact (victim only)	*n* (%)	—	
Yes	21 (50.0%)	—	
No	21 (50.0%)	—	
Tried to stop the bullying	*n* (%)	*n* (%)	0.051
Yes	26 (61.9%)	57 (43.2%)	
No	16 (38.1%)	75 (56.8%)	
Reasons for not intervening^b^	*n* (%) *n* = 16	*n* (%) *n* = 75	0.078
Avoid trouble/walk away	9 (56.2%)	25 (33.3%)	
Bully is strong/supported	5 (31.2%)	12 (16.0%)	
None of my business	0 (0.0%)	20 (26.7%)	
Already tried/futile	1 (6.2%)	5 (6.7%)	
Seemed harmless	0 (0.0%)	5 (6.7%)	
No need to	1 (6.2%)	1 (1.3%)	
Other	0 (0.0%)	7 (9.3%)	
How they intervened^b^	*n* (%) *n* = 26	*n* (%) *n* = 57	0.076
Verbally asked them to stop	15 (57.7%)	41 (71.9%)	
Asked for help from others	5 (19.2%)	8 (14.0%)	
Physically forced them	1 (3.8%)	6 (10.5%)	
Other	5 (19.2%)	2 (3.5%)	
Reported to faculty	*n* (%)	*n* (%)	1.000
Yes	10 (23.8%)	31 (23.5%)	
No	32 (76.2%)	101 (76.5%)	
Told parents or friends	*n* (%)	*n* (%)	0.461
Yes	28 (66.7%)	79 (59.8%)	
No	14 (33.3%)	53 (40.2%)	
Social support 1–5, median (IQR) (victim only)	3 (2.25, 4)	—	
Year bullying occurred (victim only)	*n* (%)	—	
1st year	20 (47.6%)	—	
2nd year	9 (21.4%)	—	
3rd year	4 (9.5%)	—	
4th year	7 (16.7%)	—	
5th year	2 (4.8%)	—	
Bullying still ongoing	*n* (%)	*n* (%)	0.838
Yes	13 (31.0%)	37 (28.0%)	
No	29 (69.0%)	95 (72.0%)	

a Multiple selections allowed; percentages may exceed 100%.

b Denominators are those who did not try/did try to stop, respectively. — Variable not applicable to this group. *p*-values from chi-square test with simulated *p*-values (one *p*-value per variable). Perpetrator labels harmonized between victim and witness questionnaires prior to comparison. Witness group includes witnesses only (*n* = 106) and victim & witness (*n* = 26). Victim group includes victims only (*n* = 16) and victim & witness (*n* = 26).

### Predictors of victimization

Logistic regression was performed to identify factors independently associated with being a victim of bullying (Table 5). Neither gender, academic major, year of study nor socioeconomic status were significant predictors. Two variables emerged as statistically significant. A history of childhood bullying was by far the strongest predictor — students with such history were over 12 times more likely to be victimized in university (OR = 12.05, 95% CI: 4.88–37.0, *p* < 0.001). Higher GPA was associated with lower odds of victimization (OR = 0.38, 95% CI: 0.18–0.79, *p* = 0.010), suggesting some protective effect of academic performance. The model fit was acceptable (AIC = 209.8) and no multicollinearity was detected (all VIF < 2). Goodness-of-fit was further confirmed by the Hosmer-Lemeshow test (*χ*^2^ = 7.456, df = 8, *p* = 0.488), indicating no significant lack of fit. The model showed good discrimination (AUC = 0.822, 95% CI: 0.763–0.873) and explained a substantial proportion of the variance in victimization (Nagelkerke R^2^ = 0.270; McFadden R^2^ = 0.209).

**Table 5:** Logistic regression: predictors of victimization (*n* = 352).

**Predictor**	**OR**	**95% CI**	***p*-value**
Gender (ref: Female)			
Male	0.92	0.40, 2.03	0.846
Major (ref: Medicine)			
Dentistry	1.12	0.53, 2.37	0.758
Year of study (ordinal)	1.30	0.97, 1.74	0.078
Socioeconomic status (ordinal)	0.99	0.59, 1.69	0.981
**GPA**	**0.38**	**0.18, 0.79**	**0.010**
**Childhood bullying history**	**12.05**	**4.88, 37.0**	**<0.001**
Single parent family (ref: Yes)			
No	0.41	0.14, 1.28	0.104

OR, odds ratio; CI, 95% confidence interval. All VIF values < 2 (no multicollinearity).

AIC = 209.8. Childhood bullying OR recalculated as inverse (ref: No childhood bullying).

Bold = statistically significant (*p* < 0.05).

Nine participants (2.3%) identified themselves as perpetrators of bullying. Given the small sample size, no inferential tests were conducted, and findings are reported descriptively only. The group was predominantly male (66.7%) with a mean age of 21.7 years (SD = 2.2). Students were distributed across Medicine (55.6%) and Dentistry (44.4%), with most in their third or fourth year of study (22.2% and 33.3% respectively). The mean GPA was 3.1 (SD = 0.3), somewhat lower than the overall sample mean of 3.4. A notable proportion were smokers (44.4% cigarettes) and the majority came from middle class backgrounds (66.7%). Two students (22.2%) were from single parent families.

A history of childhood bullying was reported by 66.7% of perpetrators, higher than the overall sample rate of 42.0%. Regarding mental health, 55.6% met the threshold for probable depression and 44.4% for probable anxiety, with median PHQ-9 and GAD-7 scores of 11 and 7, respectively. Treatment use was also notable: 22.2% were on antidepressants, 22.2% on anxiolytics, and 33.3% in psychotherapy, all considerably higher than the overall sample rates. These findings should be interpreted with caution given the very small group size. Full profile is presented in Table 6.

**Table 6:** Demographic and mental health profile of perpetrators (*n* = 9)*.

Characteristic	*n* = 9
**Demographics**	
Age, mean (SD)	21.7 (2.2)
GPA, mean (SD)	3.1 (0.3)
BMI, mean (SD)	26.0 (4.4)
Male	6 (66.7%)
Medicine	5 (55.6%)
Jordanian	8 (88.9%)
Single parent family	2 (22.2%)
Childhood bullying history	6 (66.7%)
Smoker (cigarettes)	4 (44.4%)
Drinks coffee	5 (55.6%)
**Year of study**	
2nd	2 (22.2%)
3rd	2 (22.2%)
4th	3 (33.3%)
5th	1 (11.1%)
6th	1 (11.1%)
**Socioeconomic status**	
Middle class	6 (66.7%)
Upper-middle class	2 (22.2%)
Upper class	1 (11.1%)
**Mental health outcomes**	
PHQ-9 score, median (IQR)	11 (5, 13)
Probable depression	5 (55.6%)
GAD-7 score, median (IQR)	7 (5, 11)
Probable anxiety	4 (44.4%)
**Treatment use**	
On antidepressants	2 (22.2%)
On anxiolytics	2 (22.2%)
In psychotherapy	3 (33.3%)

*Descriptive only. No inferential tests conducted due to small sample size (*n* = 9).

Probable depression = PHQ-9 ≥ 10. Probable anxiety = GAD-7 ≥ 10.

*n* (%) unless otherwise stated.

## Discussion

Higher education institutions are responsible not only for academic training but also for promoting students' mental health, well-being, and retention, which makes a safe and supportive learning environment central to their mission (22). Against this backdrop, academic bullying represents a heavy burden on medical students and trainees in terms of negative impacts on psychological aspects, self-confidence, and development of clinical skills (18). This study aimed to investigate the prevalence and characteristics of bullying among medical and dental students while focusing on key associations through a self-administered questionnaire. In general, we noted a considerable prevalence of bullying among medical students with a poor reporting rate. Significant associations were noted with childhood bullying history, GPA, severe-to-moderate anxiety disorder, and psychiatric therapies.

### Victims of bullying

Our study revealed that 11.0% of participants were victims of bullying (95% CI: 7.8–14.1%). This finding is closely similar to a very recent study in Oman, which reported a prevalence of 11.5% (5). Alternatively, the prevalence was much higher in a study in the UAE, in which 34.1% of participants were exposed to bullying (23). Additionally, a study in the USA showed that 44% of medical students had experienced mistreatment during their medical clerkships (11). However, this much higher prevalence could be due to reporting many forms of mistreatment, not just solely bullying. Data regarding prevalence in dental schools is limited in the literature. However, in one multi-national study, 34.6% of dental students reported mistreatment by their tutors/instructors, and 17% reported being bullied or mistreated by colleagues (24). In light of this, the results of our analysis demonstrated that dentistry students were slightly more likely to experience bullying than medical students. However, this difference was not statistically significant. Differences in bullying among academic majors were not clearly stated in the literature. However, one study in Saudi Arabia found that the prevalence of bullying among medical students is lower compared to non-medical students (25). Side by side, in another study in Finland, medical students reported all forms of mistreatment more than students in any other academic major (26). This may be largely influenced by the power hierarchy implanted in the medical culture (27), as workplace bullying also represents a widespread issue in residency programs and is not just exclusively limited to academic years (28). Other possible contributing factors may include increased prevalence of academic burnout and emotional exhaustion (29), along with a decline in empathy in the medical learning environment (30).

It appears from our findings that around 10% of the victims were exposed to bullying on a frequent or recurrent basis. Meanwhile, the earlier cited Omani study showed a slightly higher prevalence, as 18.2% of victims reported suffering from bullying on a daily/ weekly basis (5).

Most participants in our study were exposed to verbal bullying followed by academic, social/relational, cyber, and then discriminative bullying, respectively. Physical bullying was the least reported. These findings are largely comparable to the study by Al-Ghawi et al., which reported closely similar results (5). In addition, participants in our study commonly encountered bullying from fellow students, followed by lecturers, faculty members, then patients and their families, respectively. A similar pattern was noted in a study by Elghazally et al. (13). In contrast, participants in a study in Thailand reported mistreatment from lecturers/supervisors more than from their colleagues (31). The prominence of lecturers and supervisors among the reported sources of bullying reflects the steep power hierarchy embedded in medical training, in which the authority that instructors and senior clinicians hold over students' assessment and career progression can be misused, and such conduct may be normalised as an accepted part of the training culture (18). Further examination of the data revealed that 70% of students experienced bullying during their first and second years of study (47.6% and 21.4%, respectively). However, the year of study was not a significant predictor for victimization when performing logistic regression. Advanced-year students might be expected to report greater exposure, since cumulative opportunity increases across the academic trajectory and clinical years involve more contact with residents and attending physicians (32). In our sample, however, reported exposure was concentrated in the earlier years, suggesting that the association may reflect differences in how students at different training stages perceive and label such behaviors, rather than true differences in exposure, and that preclinical peer-to-peer dynamics may be an underrecognized source of bullying in this setting.

Social demographics, including gender, age, and socioeconomic status, were not statistically significant predictors for victimization even after applying logistic regression to control for potential confounders. This absence of a significant sex or socioeconomic difference may partly reflect Jordan's social and cultural context, where prevailing gender norms and the stigma attached to disclosing mistreatment may lead male and female students to under-report to a similar degree (33). In contrast, a study among Lebanese medical students demonstrated that female gender and insufficient family income were significant positive predictors for mistreatment in clinical rotations (34). Also, another study among Syrian medical graduates observed that females and participants under 30 years old were significantly more susceptible to bullying (35). Meanwhile, in a Pakistani study, males were significantly more exposed to bullying than females (36).

Our analysis revealed that a lower GPA was significantly associated with a higher risk for victimization among participants (OR = 0.38, 95% CI: 0.18–0.79). However, only 50% of victims reported a negative academic impact attributed to bullying. Similarly, a study conducted by El Nouiri A et al. showed that 35.4% of victims faced negative academic impact due to bullying (34). Moreover, an experimental study among medical students demonstrated a significant negative impact of mistreatment on academic achievement despite no significant differences in cognitive engagement and studying time (37). Evidence from a study by Murphy D. et al. indicated that bullying has a significant indirect association with academic achievement through emotional disturbances (38). Other factors like poor motivation, academic engagement, and self-esteem may also influence this association (39).

It was observed that median body mass index (BMI) was slightly higher among bullying victims compared to non-victims, but the difference was not statistically significant. Similarly, two studies of medical graduates have reported that BMI was not a statistically significant predictor for victimization (35, 40). In contrast, a meta-analysis of 26 cross-sectional and cohort studies exploring the influence of body weight on bullying among children and adolescents showed significantly higher risk of bullying victimization, attributed to obesity and overweight status (41). Furthermore, a multi-national study of bullying among dental students revealed that bullying victims were more likely to be worried about dieting and losing weight (24).

It can be seen from our results that smoking was lower among victims compared to non-victims, but the difference was not statistically significant. In the same context, a study among Indian medical undergraduates revealed that smoking was significantly associated with higher exposure to physical bullying, but no significant association with exposure to verbal bullying was found (42).

We noted from our analysis that participants with a positive history of childhood bullying were significantly more susceptible to bullying during college years. This aligns with a study conducted by El Nouiri et al., in which some victims reported previous exposure to bullying in primary (37.4%) and high school (32.5%) (34). Such findings may be attributed to long-term effects of childhood bullying on social isolation (43), self-dependence (2), and mental health problems (44).

To assess the psychological impact of bullying, we used PHQ-9 and GAD-7 questionnaires, 52.4% of bullying victims met the threshold for probable depression compared to 44.6% of non-victims, but this difference was not statistically significant. Probable anxiety was significantly higher among victims compared to non-victims (52.4% vs. 33.4%, *p* = 0.015). In a comparable context, where a study among medical students in Egypt used similar questionnaires, both moderate-to-severe anxiety and depression were found to be significantly higher among victims (45). Similarly, a study in Thailand, which used PHQ-9 questionnaire, demonstrated a significantly increased risk for depression among victims of different subtypes of bullying (31). Additionally, in a study by Elghazally N and Atallah A, medical students who suffer from a psychological issue were significantly more likely to experience bullying (13). In relation to the discussion, one study among dentistry students found that participants who faced bullying from fellow students were more likely to use psychiatric medications (24). This matches our findings that victims were significantly more likely to use antidepressants, anxiolytics or to receive psychotherapy than non-victims (*p* < 0.01). Based on the previous findings, it is important to develop effective mental health interventions to detect help-seeking students, as current evidence suggests very low certainty about the efficacy of existing interventions (46).

According to our findings, the responses of victims to bullying acts varied widely. Only 61.9% of them tried to stop the bully; around half of them verbally asked the perpetrator/s to stop, others sought help from others, while only one intervened physically. Among those who didn't try to stop the perpetrator/s, around half wanted to avoid trouble, some were concerned that the bully is strong or supported, and a lesser number thought it was useless or unnecessary. Moreover, a poor reporting rate for bullying events was noted, considering that some students reported poor social support. Only 23.8% of victims reported what happened to them to faculty, and 66.7% reported it to family and/or friends. Correspondingly, in a study by Ibrahim et al., 28.6% of participants didn't try to stop the perpetrator, while 11.1% defended themselves, 14.3% notified student affairs, and 46% told family and friends (23). Also, in a study assessing medical students' feedback about mistreatment in their clerkships, much worse numbers were observed as 78% of students didn't do anything about it, not even reporting the event (11). Concerning this matter, one study discussed reasons beyond hesitation to report academic violence, in which most victims stated they think it's useless, many were afraid that it would negatively affect their grades/ evaluation, while others were not sure if violence was a negative attitude or a casual norm in the medical learning process (47). Another study within the same context revealed additional causes, including difficulty reporting minor harassments and the lengthy time and big effort required for reporting (48). The preceding findings signify the need to provide proper social and academic support for medical students to limit this phenomenon, for example, a study by Ahmer S. et al. revealed a protective effect for such measurements as bullying experience was significantly related to the unavailability of adequate support (49).

### Bystanders of bullying

According to our findings, as many as 34.5% of participants have observed bullying in academic settings, with the majority of victims being friends and colleagues. One study conducted in Tanzania in 2024 showed a lower prevalence, as 28.7% of participants stated that fellow students were victims of bullying (50). Meanwhile, in two other studies, one in Syria and the other in the USA, the prevalence was much higher (87% and 61%, respectively) (35, 40).

Our data suggested that 43.2% of bystanders tried to stop the bullying act, which was relatively lower than the victims' group. However, the difference was not statistically significant (*p* = 0.051). Among those who didn't intervene, one third wanted to avoid trouble, and around one quarter think it's none of their business. Among all, 23.5% of bystanders reported the event to faculty, which is similar to the reporting rate in the victims' group. In one qualitative study discussing the roles of medical students in providing social support for peers, students have expressed some obstacles to offering help to their colleagues, including a lack of experience, poor confidence to offer support, and a lack of rapport with peers (51), further confirming the importance of providing adequate social support in medical schools.

### Engagement in bullying

Among participants in our study, only 2.3% admitted participating in bullying as perpetrators. In one study conducted in Egypt in 2022, 57.7% of participants were identified as perpetrators. Interestingly, the great majority of them were also victims (45). This study yielded a greater reporting of engagement as it didn't rely on self-reporting but rather on a questionnaire that was developed referring to the Bullying Scale for Higher Education Students (52) and on a validated Arabic version of the Olweus scale (53), which may have enhanced objectivity of reporting.

Our findings showed that at least half of the perpetrators met the threshold for probable anxiety and/or probable depression, while at least a third of them used anxiolytics, antidepressants, and/or received psychotherapy. This matches a meta-analysis conducted by Ye Z et al., in which a significant risk of depression among perpetrators was established (54). In addition, we found two thirds of perpetrators were subjects of bullying in their childhood. This also matches the results of Jankowiak B. et al., that exposure to physical and or sexual abuse in addition to low social support from colleagues, was associated with a significant risk for becoming a perpetrator (55). Beyond that, another study highlighted the modulating effects of anxiety and poor social skills on the association between childhood bullying and becoming a perpetrator (56). Accordingly, in order to prevent the bullying process, social and psychological support should also be provided for perpetrators and not just limited to victims.

Understanding bullying around the world and using a public health approach to address it is at the heart of the Sustainable Development Goal (SDG) target 4.a, which aims to broadly track the overall school environment beyond pedagogical matters. Indicator: 4.a.2 Percentage of students experiencing bullying, corporal punishment, harassment, violence, sexual discrimination and abuse (57). Bullying carries significant negative repercussions for bullies, victims, and witnesses. Multilayer Interventions are recommended to improve school climate including lowering competition and enhancing involvement family and peers (58). Academic bullying goes beyond the scope of this research; future research is needed to address bullying dynamics and misconduct in scientific and scholarly publishing. Although authors' integrity has been extensively studied, there is still a gap in literature concerning editor's role in safeguarding the rigor and credibility of published research (59). Transparency in the editorial process must extend beyond authorship disclosures to include any practices that could compromise ethical norms (60).

## Strengths and limitations

This study explores an important and under-researched issue of academic bullying among medical and dental students. Our study is the first to investigate this topic in Jordan, as previous research in the country has focused on other academic majors and school-aged populations. We also examined the phenomenon from multiple angles by exploring victims, bystanders, and perpetrators' perspectives, which allowed for direct comparison of behavioral responses and reporting patterns across these groups. Besides that, the use of logistic regression with adjustment for seven potential confounders strengthened the reliability of the associations identified. Nonetheless, residual confounding from unmeasured variables, such as personality traits, family dynamics, or prior exposure to violence outside the academic setting, cannot be excluded. The cross-sectional design, however, does not permit causal or temporal inferences. The direction of the association between GPA and victimization, for example, cannot be determined from these data, as lower academic performance may either precede or result from bullying exposure. The same ambiguity applies to the relationship between anxiety and victimization (18).

The sample of our study was drawn from a single institution using convenience sampling, which limits generalizability to other academic settings. Moreover, because participants were recruited online through official student WhatsApp and Facebook groups, self-selection may have introduced a degree of selection bias, as students who were less active on these platforms or less interested in the topic may have been underrepresented. Furthermore, because the questionnaire was distributed openly through these online groups rather than to a fixed, enumerated list of invitees, the total number of students who viewed the invitation could not be tracked, and a conventional response rate could not be calculated. This open distribution format also precluded technical safeguards against duplicate submissions from the same respondent, although the anonymous, unincentivized nature of the survey provided little motive for repeat participation. The small number of victims (*n* = 42) and perpetrators (*n* = 9) may have limited statistical power, and findings on perpetrators should be interpreted as descriptive only.

While depending on online questionnaires for data collection may have enhanced reliability of reporting by promoting privacy, it could have introduced misunderstanding of some questions. In addition, depending on self-report may introduce the risk of recall and social desirability bias, which may have amplified underreporting. This concern is particularly relevant for perpetrator identification. Recall bias is also a concern for the childhood history of bullying, as this relied on participants retrospectively recalling experiences from many years earlier, which may have led to misclassification of prior exposure.

## Recommendations

Despite the prevalence of bullying in this study being relatively low compared to existing literature, it continues to reflect on a significant issue that calls for effective interventions which mainly should focus on improving social behaviors of medical students through educational workshops and awareness campaigns, while at the same time providing adequate training for lecturers and supervisors that addresses their academic roles in respectful supervision and the boundary between constructive feedback and bullying, particularly given the influence of power hierarchies in sustaining bullying behavior in medical settings (18, 27). Moreover, the low reporting rate of bullying indicates that medical schools should establish confidential, structured reporting channels that protect students' privacy while taking deterrent actions against perpetrators.

Childhood bullying was the strongest predictor of victimization in the regression model. Screening incoming students for prior adverse experiences during academic orientation may help buffer against revictimization. Additionally, this finding states the importance of the earlier implementation of bullying controlling policies in school environments. The majority of bullying incidents started during the first two years of college, which also suggests that interventions should be concentrated early in the curriculum.

The psychological impacts of bullying support the integration of periodic mental health screening. Access to mental health services should be expanded and destigmatized, given the low certainty around the efficacy of current help-seeking interventions among medical students (46). The data on perpetrators also showed elevated rates of childhood bullying, depression, and psychiatric treatment use. Thus, interventions should therefore not be limited to victims, as psychological support and restorative approaches for perpetrators may help break the cycle of bullying.

Prevention efforts should also draw on institutional policies and interventions that have shown promise internationally. Comprehensive, clearly disseminated anti-bullying policies and codes of conduct, coupled with confidential and trusted reporting mechanisms, are foundational, as weak enforcement of institutional policies has been identified as a key facilitator of academic bullying (4, 18). Faculty and staff development on professional behaviour and feedback, bystander-intervention and peer-support training, structured mentorship, and multidisciplinary oversight committees that engage all stakeholders have been proposed as practical measures to foster a safer and more supportive learning environment (61). Adapting such measures to the Jordanian context could help reduce the occurrence of bullying identified in this study.

Future research should use longitudinal designs to establish temporal relationships between bullying exposure and outcomes, multi-center samples to improve generalizability, and validated behavioral scales rather than self-labeling to estimate perpetration more accurately. Mixed-methods approaches may offer deeper insight into cultural barriers to disclosure. Similarly, studies evaluating specific anti-bullying programs in medical schools remain scarce and needed (18, 46).

## Conclusion

This cross-sectional study aimed to investigate the prevalence of bullying and its patterns in the settings of medical education in Jordan while assessing psychological and academic impacts, along with key associations with this phenomenon. The results showed that a fair proportion of participants reported being subjected to and/or bystanding bullying during their academic years, most commonly from their colleagues. Meanwhile, a tiny proportion reported being perpetrators. Victimization was associated with negative psychological and academic outcomes as well as childhood experience of bullying. Finally, this study has exposed a poor rate of reporting of bullying incidents, denoting the importance of addressing bullying in medical education by establishing accessible reporting systems, routine mental health screening, and peer-support programs. Longitudinal and multi-institutional studies are needed to clarify causal pathways and evaluate the effectiveness of such interventions.

## Data Availability

The raw data supporting the conclusions of this article will be made available by the authors, without undue reservation.
